# Breastfeeding policy and practices at the general paediatric outpatient clinic of a teaching hospital in Lagos, Nigeria

**DOI:** 10.1186/1746-4358-9-10

**Published:** 2014-06-27

**Authors:** Idowu O Senbanjo, Kazeem A Oshikoya, Okeoghene A Ogbera, Kikelomo O Wright, Alexandra L Anga

**Affiliations:** 1 Department of Paediatrics and Child Health, Lagos State University College of Medicine, PMB 21266 Ikeja, Lagos, Nigeria; 2 Department of Pharmacology, Lagos State University College of Medicine, Ikeja, Lagos, Nigeria; 3 Department of Internal Medicine, Lagos State University College of Medicine, Ikeja, Nigeria; 4 Department of Community Health and Primary Health Care, Lagos State University College of Medicine, Ikeja, Nigeria

**Keywords:** Breastfeeding, Policy, Practices, Out-patient clinic, Nigeria

## Abstract

**Background:**

Hospitals have a role to play in supporting, protecting and promoting breastfeeding. The aim of this study was to describe hospital breastfeeding policy and practices and breastfeeding rates among mothers attending General Paediatric Outpatient Clinic at a tertiary hospital in Lagos, Nigeria.

**Methods:**

This was a cross-sectional study involving paediatric nurses and doctors, as well as the mothers who brought their child to the General Paediatric Outpatient Clinic. Two sets of questionnaires, different in content, were administered to doctors and nurses, and to mothers of children aged 6-24 months, to assess hospital policy and breastfeeding rates, respectively. Stepwise multiple logistic regression analysis was used to examine factors associated with duration of breastfeeding.

**Results:**

Although the hospital had a written breastfeeding policy copies of the policy were not clearly displayed in any of the units in the Paediatric department. Almost half the staff (48%; 60/125) were not aware of the policy. The hospital had no breastfeeding support group. Nearly three quarters (92/125) of the staff had received lactation management training. 36% (112/311) of mothers exclusively breastfed for six months, 42% (129/311) had stopped breastfeeding at the time of the survey. 67% (207/311) of babies were given infant formula, 85% (175/207) before 6 months. Women who had antenatal care in private hospitals and were Christian were more likely to breastfeed exclusively for 6 months. Low maternal education was the only factor associated with breastfeeding longer than 12 months.

**Conclusion:**

Breastfeeding practices and policy implementation at this outpatient clinic were suboptimal. We have identified a need for interventions to increase knowledge of the benefits of breastfeeding and to provide support for its longer term duration. We suggest that BFHI be considered across all facilities concerned with infant and early child health to disseminate appropriate information and promote an increase in exclusive breastfeeding for six months as well as the duration of breastfeeding.

## Background

Evidence shows that breastfeeding for the first two years of life has numerous benefits for mother and child [1-6]. Breastfeeding reduces morbidity and mortality from common childhood diseases such as diarrhoea, acute respiratory infections, and protein energy malnutrition [2,3]. It also allows for proper maternal and child bonding and offers mothers some protection against ovarian cancer, breast cancer and hip fractures [4-6]. However, this natural practice is not universally embraced in both developed and developing countries, the rates of exclusive breastfeeding for the first six months of life and breastfeeding up to the age of two years are alarmingly low [7]. As a result, the United Nations Children International Funds (UNICEF) and the World Health Organization (WHO) launched the Baby Friendly Hospital Initiative (BFHI) in 1991 [8]. The main objective was to mobilize health care systems and health workers to promote, protect and support breastfeeding practices. Since inception of the BFHI, 21,328 facilities in 131 countries have been accredited as baby friendly hospitals [9]. Cross-sectional surveys from the different geo-political zones in Nigeria recorded the prevalence of exclusive breastfeeding for the first six months of life ranging between 19-30%. Similarly, in a recent National Demographic and Health Survey in Nigeria, the rate of exclusive breastfeeding for the first six months of life has actually dropped by 4% (from 17% in 2003 to 13% in 2008) [10]. In order to achieve the target rate of 90% for exclusive breastfeeding by the year 2015 [11], efforts at improving breastfeeding practices need to be sustained, and continuous campaigns on the part of the government and other stakeholders need to be intensified. The current practice in Nigeria is for BFHI activities to be concentrated in maternity, post-natal and, well-baby clinics and primary health care and immunization centres. Other potential channels such as the General Paediatrics Outpatient Clinic (GPOC) that could be used to educate and motivate mothers about breastfeeding practices are rarely utilized [12]. Although the 2015 deadline to achieve exclusive breastfeeding globally approaches, actualisation of the BFHI targets in many countries remains beyond reach. Evaluation and reappraisal of BFHI activities is needed to strengthen breastfeeding practices among mothers attending paediatric outpatient clinics of all public hospital in Nigeria.

Lagos State University Teaching Hospital (LASUTH) provides paediatric and child health services to a considerable number of inpatients and outpatients and is one of the accredited BFHI centres in Nigeria.

The aims of this study were to describe hospital breastfeeding practices and policy at the GPOC; describe the breastfeeding rates for mothers attending and to determine the factors affecting the duration of exclusive breastfeeding.

## Methods

### Setting

This was a prospective cross-sectional study carried out at the GPOC, LASUTH. The hospital is a tertiary health facility owned by the Lagos state government. Located in Ikeja local government area, the hospital serves the inhabitants of Lagos and the neighbouring Ogun state and provides subsidized health care for children and the elderly.

The Paediatric department employs 11 Consultant Paediatrician, one a locum,, 45 other doctors (comprising 30 residents, 5 medical officers and 10 contract medical officers) and 84 nurses. Staff rotate through the GOPC on a monthly basis and the service is usually staffed by a consultant paediatrician, eight doctors and ten nurses. The clinic is open every day and attended by an average of 90 patients daily. Breastfeeding practices and policy at the GPOC were descibed by assessing the presence or otherwise of a written breastfeeding policy and breastfeeding support groups, both believed necessary to promote breastfeeding practice. We used a questionnaire to survey the opinions of clinical staff about whether there was a breastfeeding policy or there were any breastfeeding support groups in the hospital. Information about individual staff participation in breastfeeding education and roles in supporting hospital based breastfeeding activities were also sought.

Mothers with children aged 6–24 months who attended the GOPC, between 1 July and 31 August 2012, were approached consecutively, those who agreed to participate were recruited. Questions sought information about breastfeeding practices and relevant factors pertinent to the index baby. Interviews were completed by medical officers who were briefed about the interview process and utilised a questionnaire specifically designed for the study. Relevant background information including infant and parental age, education, parity and any previous breastfeeding was obtained in addition to socio-economic status, and current breastfeeding practice. Families were classified according to socio-economic class using Ogunlesi et al. [13]. Using this system of classification, occupation and highest educational attainment of each parent were scored descending from 1 to 5. The parental mean score determined classification. Those with a mean score of 1 or 2 were further re-classified as upper class, while those with mean scores of 3, 4 and 5 were re-classified as lower social class. Upper social class includes parents such as senior public officers, large scale traders, large scale farmers and professionals while lower social class are peasant farmers, artisans and labourers.

### Ethical issues

Ethical approval was obtained from the Lagos State University Teaching Hospital Research and Ethics Committee, reference number LREC/10/06/378. Written informed consent was obtained from each respondent.

### Definition of terms

***Ever breastfed***: The child was ever breastfed.

***Exclusive breastfeeding****:* The child was breastfed exclusive of any other food or fluid with the exception of small amounts of medicinal supplements.

***Predominant breastfeeding****:* When breast milk was the main source of nourishment but supplemented by water, water based dinks, fruit juice, drops or syrups containing vitamins and minerals, supplements or medicines. Other food-based fluids, such as non-human milk, were excluded.

***Pre-lacteal feeds***: Introduction of fluid or feeds prior to lactation.

### Statistical analysis

Data analysis was by descriptive and inferential statistics using SPSS for Windows software version 11. Univariate analyses were performed for all major variables of interest, such as demographic, socio-economic status, breastfeeding and formula feeding. Mean and standard deviations (SD) were determined for continuous variables while ratios and proportions were calculated for categorical variables. The prevalence estimate for exclusive breastfeeding for the first 6 months of life and any breastfeeding for 12 months were defined using specific socio-demographic characteristics to identify potential risk factors for exclusive breastfeeding for 6 months and any breastfeeding for 12 months. Logistic regression using stepwise analysis was used to determine the predictive variables for exclusive breastfeeding for the first six months of life and any breastfeeding for 12 months after adjusting for confounding variables. A p-value of less than 0.05 was accepted as statistically significant.

## Results

Eighty nine percent (125/140) of the paediatric staff returned completed questionnaires. All 311 mothers who met study inclusion criteria consented to participate and completed interviews.

### Characteristics of GPOC and staff

The hospital has a written hospital policy on breastfeeding practices, but none was posted on the wall at the GPOC or any of the other units in paediatrics department. There was no existing breastfeeding support group for mothers in paediatric department. Of the 125 staff, 65 (52%) were aware that the hospital has a written policy that supports breastfeeding. Ninety-two (73.6%) staff had ever received lactation management training, only 2 (1.6%) had been trained in the last year. The duration of training ranged from 3 to 5 days. Nineteen (23.5%) staff were involved with assisting mothers (counselling and teaching) on how to breastfeed in the last three months.

### Sociodemographic characteristics of mothers and children

Table 1 shows the socio-demographic characteristics of the mothers and their children. The age of the mothers ranged from 12 to 49 years, with mean age of 31.2 years (SD 5.3). Most of the mothers (90.7%) had at least secondary education and were married (96.8%). About half (48.6%) of the mothers had their antenatal care in a private hospital. One hundred and sixty-six (53.4%) mothers gave birth in a private hospital.

**Table 1:** Socio-demographic characteristics of mothers anf children

**Parameters**	**No. (n = 311)**	**%**
**Age of mother**		
≤ 30 years	153	49.2
> 30 years	158	50.8
**Age of father**		
≤ 30 years	40	12.9
> 30 years	271	87.1
**Age of children (months)**		
6 - 11	139	44.7
12 - 17	80	25.7
18 - 24	92	29.6
**Place of residence**		
Rural	24	7.7
Urban	287	92.3
**Eductional level of mothers**		
No formal education	2	0.6
Primary	27	8.7
Secondary	122	39.2
Tertiary	160	51.4
**Parity**		
**1**	81	26.0
**2**	106	34.1
**≥ 3**	124	39.9
**Religion**		
Christian	241	77.5
Muslim	68	21.9
Other	2	0.6
Place of ante-natal care*		
Private hospital	151	48.6
Tertiary hosptal	15	4.8
General hospital	66	21.2
Primary health care centre	48	15.4
Traditional birth attendant home	5	1.6
Mission home	19	6.1
Home	1	0.3
**Place of delivery**		
Private hospital	166	53.4
Tertiary hosptal	10	3.2
General hospital	60	19.3
Primary health care centre	31	10.0
Traditional birth attendant home	5	1.6
Mission home	27	8.7
Home	12	3.9

*Total number of mothers who had antenatal care is 305.

### Feeding practices and breastfeeding rates

Ninety seven percent (300/311), breastfed their babies after birth. 27% (81/311), initiated breastfeeding within one hour and 54% (162/311), within 6 hours of the birth. Thirty six percent (112/311), of infants were exclusively breastfed for six months. Two hundred and seven (66.6%) children were given infant formula feeds, of which 175 (84.5%) children were introduced to infant formula feeds before the age of 6 months (Table 2). One hundred and twenty-nine mothers (41.5%) had stopped breastfeeding. Breastfeeding duration ranged between 1 and 19 months. The mean duration of breastfeeding was 11.5 months (SD 4.2). Table 3 shows the reasons why mothers stopped breastfeeding their children before the age of 24 months. About half of the mothers (47.3%) stopped breastfeeding because they thought the duration that they had achieved was adequate.

**Table 2:** Feeding practices and breastfeeding rates

**Parameters**	**No. (n =311)**	**%**
Prelacteal feeds	166	53.4
Ever breastfed	300	96.5
Initiation of breastfeeding:		
< one hour	81	26.0
one - < 6 hours	81	26.0
6 - < 24 hours	83	26.7
≥ 24 hours	66	21.2
Exclusive brestfeding for six months	112	36.0
Predominantly breastfeding for six months	36	11.6
Formula feeding	207	66.6
Breastfeeding at the time of survey:		
6 -11 months	126	90.6
12 -17 months	47	58.8
18 -24 months	9	9.8

**Table 3:** Reasons for cessation of breastfeeding (n = 129)

	**No.**	**Percentage**	**Mean (SD) age of cessation of breastfeeding**
Maternal decision	61	47.3	13.6 (3.1)
Baby stopped breastfeeding	21	16.3	9.8 (3.3)
Maternal occupation	14	10.9	9.7 (4.7)
Maternal illness	8	6.2	12.1 (3.2)
Pregnancy	8	6.2	11.8 (2.3)
Not enough milk	4	3.1	8.0 (5.8)
Baby biting mother	3	2.3	14.3 (3.2)
HIV positive mother	3	2.3	1.3 (0.6)
Breastfeeding too demanding/tiring	3	2.3	14.7 (0.6)
Advice from peers	2	1.6	14.5 (0.7)
Loss of husband	1	0.8	12
Twin delivery	1	0.8	10

### Factors associated with exclusive breastfeeding and duration of breastfeeding

Tables 4 and 5 show the factors associated with exclusive breastfeeding and duration of breastfeeding, respectively. Christian background (OR 3.0; 95% CI 1.7, 5.2; p < 0.001) and antenatal care in private hospitals (OR 3.8; 95% CI 1.2, 11.5; p = 0.029) were significantly associated with exclusive breastfeeding, while low maternal education (OR 3.0; 95% CI 1.4, 6.1; p = 0.003) was the only factor identified to be associated with breastfeeding duration longer than 12 months. Results of the multiple regression analysis for exclusive breastfeeding for six months and any breastfeeding for 12 months are presented in Table 6. After adjusting for potential confounders, antenatal care in private hospitals and Christian background were significant determinants of exclusive breastfeeding explaining 6.8% of the variance. Low maternal education remains statistically significant in relation to breastfeeding beyond 12 month of age contributing about 4% of the variance.

**Table 4:** Factors associated with exclusive breastfeeding for six months (n =311)

**Parameters**	**Exclusive breastfeeding**				**Odds ratio**	**95% CI**
	**Yes**		**No**			
	**No.**	**%**	**No.**	**%**		
Maternal age						
≤ 30 years	55	35.9	98	64.1	1	
> 30 years	57	36.1	101	63.9	1.01	0.6, 1.6
Infant sex						
Female	42	32.8	86	67.2	1	
Male	68	37.2	115	62.8	1.2	0.8, 1.9
Parity						
Multiparity	79	34.5	150	65.5	1	
Primiparity	33	40.2	49	59.8	1.3	0.8, 2.1
Religion						
Muslim	19	18.5	75	81.5	1	
Christian	93	42.9	124	57.1	3.0	1.7, 5.2*
Maternal education						
At least secondary	48	31.8	103	68.2	1	
Postsecondary	64	40.0	96	60.0	1.4	0.9, 2.3
Social class						
Upper	27	34.6	51	65.4	1	
Lower	85	36.5	148	63.5	1.1	0.6, 1.9
Place of antenatal care						
Private hospital	63	41.7	88	58.3	3.8	1.2, 11.5*
Government hospitals	40	31.0	89	69.0	2.2	0.8, 7.3
Others^†^	4	16.0	21	84.0	1	
Place of delivery						
Private hospital	65	39.2	101	60.8	1.0	0.5, 2.0
Government hospitals	30	29.7	71	70.3	0.7	0.3, 1.4
Other^†^	17	38.6	27	61.4	1	

*p <0.05; ^†^Other - homes, mission homes and traditional birth attendance.

**Table 5:** Factors associated with any breastfeeding at 12 months (n=129)*

	**Duration of breastfeeding (months)**				**Odds ratio**	**95% CI**
	**≤ 12**		**> 12**			
**Parameters**	**No.**	**%**	**No.**	**%**		
Maternal age (Years)						
> 30	38	53.5	33	46.5	1	
≤ 30	24	41.4	34	58.6	1.6	0.8,3.3
Child sex						
Female	27	49.1	28	50.9	1	
Male	35	47.3	39	52.7	1.1	0.5,2.2
Parity						
Multiparity	44	50.0	44	50.0	1	
Primiparity	18	43.9	23	56.1	1.3	0.6,2.7
Religion						
Muslim	9	32.1	19	67.9	1	
Christian	53	52.5	48	47.5	0.4	0.2,1.0
Maternal education						
Post secondary	43	59.7	29	40.3	1	
At least secondary	19	33.3	38	66.7	3.0	1.4,6.1^†^
Social class						
Upper	18	60.0	12	40.0	1	
Lower	44	44.4	55	55.6	1.9	0.8,4.3
Place of antenatal care						
Private hospital	31	58.3	29	41.7	1.0	0.5-,.1
Government hospitals	31	51.9	30	48.1	1	
Others^‡^	0	0.0	7	53.8	Undefined	Undefined
Place of delivery						
Private hospital	35	48.6	37	51.4	1.7	0.5,5.6
Government hospitals	22	51.2	21	48.8	1.9	0.5,6.6
Other^‡^	5	35.7	9	64.3	1	

*The other 182 children are currently been breastfed; ^†^p < 0.05; ^‡^Other - homes, mission homes and traditional birth attendance.

**Table 6:** Multiple regression analysis of factors associated with exclusive breastfeeding for 6 months and any breastfeeding for 12 months

**Dependent variable**	**Independent variable**	**R**	**r**^**2**^	**F**	**Sig. F change**
Exclusive breastfeeding for 6 months^a^	ANC in private hospital	0.217	0.047	10.8	0.001
	Christian religion	0.261	0.068	8.0	0.028
Breastfeeding beyond 12 months^b^	Low maternal education	0.207	0.040	7.6	0.006

ANC - antenatal care; low maternal education - equal to less than secondary education; ^a^Adjusted for maternal age, parity, maternal education, social class, place of delivery and child sex; ^b^Adjusted for maternal age, parity, religion, social class, place of ANC, place of delivery and child sex.

## Discussion

This study described breastfeeding policy and practices in addition to breastfeeding rates and duration amongst a group of mothers who attended a BFHI accredited paediatric clinic in Lagos, Nigeria. Ninety seven percent of mothers initiated breastfeeding and 91% were continuing to breastfeed twelve months later. Although breastfeeding rates and duration amongst these study participants are commendable, the suboptimal duration of exclusive breastfeeding in the first six months is of concern. The 36% exclusive breastfeeding rate at six months observed in our study was similar to the 33% reported in a Baby Friendly Hospital in Enugu, Nigeria [14]. The rate was also similar to the 37% reported among the nursing mothers attending a comprehensive health centre in Nnewi, Nigeria [15] and the 30% reported in a community in Sokoto state, Nigeria [16]. However, the rate was higher than the 19% reported in an urban comprehensive health centre in Ile-Ife, Southwest Nigeria [17] and the 13% obtained from a national demographic health survey in Nigeria [10]. Although, there are discrepancies in the exclusive breastfeeding rates reported in the various regions in Nigeria, the reported rates are too low compared to the set target for 2015 of 90% recommended by the WHO [11]. These results suggest a need for education to raise community, health professional and maternal awareness about the importance of breastfeeding, particularly exclusive breastfeeding in the first six months.

The low rate of exclusive breastfeeding observed in our study, as well as the low rates previously reported in Nigeria, was in contrast to the situation reported in the neighboring West African countries. For instance, in Ghana, exclusive breastfeeding rate was 63%, while in Togo, exclusive breastfeeding rate increased from 48% in 2011 to 63% in 2012 [18]. The low rates of exclusive breastfeeding practices in Nigeria may have contributed to the high burden of under-nutrition and high mortality rate among Nigerian children when compared to Ghana [18] and Togo [18].

This is the first documented description of BFHI activities in LASUTH and it is therefore difficult to make an assertion on the trends of breastfeeding practices, rates and policy in this hospital. Evaluation of BFHI accreditation in many regions of the world has demonstrated the intervention as an important strategy for promoting, protecting and supporting breastfeeding. Ojofeitimi and colleagues in Ile-Ife, Nigeria [19] demonstrated an increase in the breastfeeding rates among mothers attending a BFHI accredited centre compared to those attending another non-accredited one. Our findings reveal that current BFHI activities in our hospital would benefit from attention to breastfeeding promotional campaigns that have been successful in other facilities.

Every breastfeeding mother who visits our hospital presents an opportunity for health education. The prevalence of *Predominant* rather than *Exclusive* breastfeeding and the introduction of formula feeding identified in our study presents a challenge. Alerting our staff, mothers and the wider community to the risks of gastroenteritis, nutritional deficit and their consequences is imperative. The establishment of an active breastfeeding support group to provide education and support for breastfeeding is one BFHI strategy deserving attention at our clinic. Prominent signage of our breastfeeding policy is another. Previous studies have shown an improvement in the practice of exclusive breastfeeding after reinforced education of the mothers [20]. Providing breastfeeding information when mothers visit our outpatient clinic may reinforce information given in hospital.

We found higher exclusive breastfeeding amongst women with a Christian background and those who had their antenatal care at private hospitals. These findings are similar to other studies which reported that Christian religion was associated with a number of healthy behaviors [21-23].

In contrast to our findings, a study in Ibadan, southwest Nigeria found that mothers who delivered at a tertiary or secondary health facilities were more likely to breastfeed exclusively for six month compared to the mothers who delivered in private hospitals [24]. This difference may be due to a decline in the promotion of breastfeeding activities in government secondary and tertiary hospitals [25].

The duration of breastfeeding has an influence on a child’s nutritional status, morbidity and mortality [26]. WHO recommends breastfeeding up to and beyond 2 years [27]. In our study, most mothers breastfeed up to the age of 12 months, thereafter, breastfeeding rates gradually decline. Many of the reasons given for earlier cessation of breastfeeding highlight areas where we need to support mothers. These reasons need to be addressed during promotional campaigns aimed at encouraging breastfeeding. Similar to other studies [28,29], we found an association between a higher level of education and earlier cessation of breastfeeding. This may be due to the fact that mothers with high level of education are career women who have to return back to work due to a short maternity leave, lack of electricity to preserve expressed breast milk, and lack of company policies that allow the establishment of a crèche within the company where mothers can take a break to breastfeed their children. Our findings contrast those from a similar study in Nairobi, Kenya [30] which demonstrated that mothers with higher education levels breastfed for longer duration than those with a lower level of education. This difference was ascribed to relatively higher prevalence of HIV infection among those with lower education in the Kenyan subjects which may be associated with early cessation of breastfeeding.

Our study findings demonstrate a need to provide better breastfeeding education and support at every opportunity. This is especially important in a country like Nigeria where poverty, malnutrition and diarrheal diseases remain prevalent among infants.

Our findings also suggest that focus should be on exclusive breastfeeding for the first six months of life and increasing duration of breastfeeding by mothers. Thus, the goal of interventions should include increasing knowledge on long-term benefits of breastfeeding and the provision of long term breastfeeding support for mothers.

The limitation of this study is that it is cross sectional and therefore caution must be exercised in making causal influence of the identified determinants on exclusive breastfeeding for six months of life and total duration of breastfeeding. Another limitation is the small sample size of mothers which may have resulted in many important independent variables that were not significant. We proposed that future research studies with a larger sample size compare breastfeeding practice at the GOPC with like services to identify effective strategies to promote, protect and support breastfeeding.

## Conclusion

In conclusion, breastfeeding practices and BFHI policy implementation at LASUTH GPOC was suboptimal. The attitude of mothers towards breastfeeding was good but the rate of exclusive breastfeeding for six months and breastfeeding up to the age of two years was very low because of the introduction of other foods, in the former instance and earlier than optimal cessation of breastfeeding in the latter. We suggest that BFHI initiatives be considered across all facilities concerned with infant and early child health to disseminate appropriate information and promote an increase in exclusive breastfeeding for six months as well as the duration of breastfeeding.

## Competing interests

The authors declare that they have no competing interests.

## Authors’ contributions

IOS conceived and design the study, supervised data collection, analyse and wrote the first draft. KAO participated in the design of the study, analysis of data and writing the first draft. OAO participated in the design, supervision of data collection and analysis of data. KOW participated in data analysis and interpretation of results and ALA participated in interpretation of results and writing of the first draft. All authors read and approved the final draft.
